# Don't Judge a Book by Its Cover: Factitious Disorder Imposed on Children-Report on 2 Cases

**DOI:** 10.3389/fped.2018.00110

**Published:** 2018-04-18

**Authors:** Noemi Faedda, Valentina Baglioni, Giulia Natalucci, Ignazio Ardizzone, Mauro Camuffo, Rita Cerutti, Vincenzo Guidetti

**Affiliations:** ^1^Section of Child and Adolescent Neuropsychiatry, Department of Human Neuroscience, Sapienza University of Rome, Rome, Italy; ^2^Child and Adolescent Neuropsychiatric Unit, Azienda USL Toscana Sudest, Grosseto, Italy; ^3^Department of Dynamic and Clinical Psychology, Sapienza University of Rome, Rome, Italy

**Keywords:** factitious disorder, münchausen syndrome by proxy, child abuse, neglect, children, perpetrator, psychiatric disorder

## Abstract

Factitious Disorder Imposed on Another (FDIA), also known as Munchausen Syndrome by Proxy (MSbP) is a very serious form of child abuse. The perpetrator, usually the mother, invents symptoms or causes real ones in order to make her child appear sick. Usually this is due to a maladaptive disorder or to an excessive of attention-seeking on her part. We report here two new cases of FDIA. The first one is a 9-year-old boy with a history of convulsive episodes, reduced verbal production, mild psychomotor disorder and urological problems who underwent several invasive procedures and hospitalizations before a diagnosis of FDIA was made. The second is a 12 year-old girl with headache, abdominal pain, lipothymic episodes, seizures and a gait impairment, who was hospitalized in several hospitals before an FDIA was diagnosed.

## Background

In the past Factitious Disorder Imposed on Another (FDIA) in children was referred to as Munchausen syndrome by proxy (MSbP). The new definition was coined because it describes a behavioral pattern rather than an underlying psychiatric syndrome, and it therefore is more accurate than MSbP [1–3]. FDIA is a relatively rare but an increasing and severe form of child abuse. Studies reported a mortality rate between 6 and 10% of all FDIA victims, making it one of the most lethal forms of abuse [4, 5].

Carter et al. [6] described the disorder as “an often-misdiagnosed form of child abuse in which a parent or caregiver, usually the mother intentionally creates or exaggerates an illness in order to keep the child in prolonged contact with health providers.” The syndrome is difficult to detect although it presents specific features. The characteristics of caregivers and of FDIA victims are reported on Tables 1, 2 [4, 7–11].

For a long time, there was a huge debate around this disorder, which lacked a common nomenclature [11]. The DSM-III (1980) and the DSM-III-R (1987) listed the term Munchausen Syndrome (MS) but not that of MSbP. The DSM-IV (1994) and the DSM-IV-TR (2000) later proposed the classification of MSbP, which was finally recognized as a distinct disorder in DSM-5 [12] as a subtype of Factitious Disorders.

According to DSM 5, the following criteria must be met in order to make the diagnosis of FDIA:

- The Perpetrator engages in the deceptive falsification of physical or psychological signs or symptoms, or of induction of injury or disease in another;
- The Perpetrator presents the victim to other as ill, impaired or injured;
- The deceptive behavior is present also in absence of external incentives (e.g., in the case of malingering);
- The behavior is not better accounted for by another mental disorder (e.g., psychotic or delusional disorder).

We report on two cases of this relatively rare but very serious form of child maltreatment, representing expressions of this disorder at different childhood ages. Our aim is to shed light on the assessment, the diagnosis, and the management of this condition, giving also an overview on common and peculiar features of both perpetrators and of victims. These descriptions might contribute to draw a specific profile of these situations thus helping clinicians in making the diagnosis of FDIA.

## First case report

### The victim

M., a 9 years-old boy, was taken to a hospital in Northern Italy by his mother for the first time at the age of 15 months following a car crash. The mother reported he suddenly stopped walking and talking. He started again around the age of 2 years, with a big delay in the normal developmental milestones. At the age of 25 months M. was admitted to the hospital for the second time for gait impairment and night-time convulsive episodes. The elettroencephalogram (EEG) exam did not show evidence of any pathological elements. Two months later he was brought to the hospital for a medical evaluation and he was diagnosed as having a mild psychomotor disorder and a severe deficit in speech development.

After M.'s father abandoned the family when the child was three, the mother reported that the child had convulsive episodes during his sleep and episodes of altered consciousness with confusion. During the third hospitalization the child did not present any seizure or other symptom. The child was discharged with a diagnosis of microcephaly, mild neuromotor delay, microcytic anemia and IgA deficiency. A few months later M. was hospitalized for the fourth time and the discharge report mentioned a normal speech development, a normal IQ and undefined paroxysmal episodes during the sleep. After a few weeks, M. was hospitalized again for the fifth time, undergoing a first invasive surgical procedure with a marsupialization of an arachnoid cyst in the left temporal lobe. He was discharged with a diagnosis of temporal cyst epilepsy and started a therapy with Tegretol. M. began to show a reduced verbal production, but none of the planned speech therapy sessions were attended by the child and the mother. M. had successively been admitted to another hospital for the 6th time on the basis of neurological absences reported by the mother, which however were never observed by M.'s teachers in school. He was classified as disabled from the National Insurance System, with a diagnosis of absence epilepsy and neuromotor delay. Later, M. was hospitalized for the 7th time, but all medical investigations were negative: the laboratory findings, the EEG exams, the Nuclear Magnetic Resonance (NMR), the neuropsychiatric evaluation and the genetic analysis were completely normal. The symptoms reported by the mother were inconsistent with the clinical observations and investigations.

The boy was hospitalized for the 8th time with a second surgical procedure of circumcision. Moreover, the mother reported that M. experienced sleep disorders with somnambulism, sleep terrors, and frequent awakenings. These disturbs were never observed or detected by the medical staff.

Subsequently, M. presented fractures of teeth; the mother accused and sued the school for the incident, but the teachers denied the occurrence of any injury during school activity. Additionally, the child confirmed that he had broken his teeth while at home. The boy was referred to the hospital for the 9th time, and the clinical report definitely stated that M. was healthy. Notwithstanding, the mother reported several urological problems after the circumcision and another surgery (third surgery) was required.

After the unclear injury with teeth fracture, the CNP requested a meeting with the teachers. They reported that M. had a normal cognitive level, with good memory and learning abilities and no concentration or attention problems. The child was reported as lively, sociable and self-sufficient, showing no defiant or aggressive behavior. However occasional inappropriate and infantile behaviors were described, which were deemed to be linked to a difficult situation at home.

### The perpetrator

During all hospitalizations, the mother stayed in the hospital for prolonged periods, caring for the child. She showed over-concern about the child's illness and she repeatedly requested further medical investigations, however she did not show any emotional involvement. For a long time she maintained a cooperative relationship with the medical and paramedical staff, which however deteriorated, turning into a conflictual relation when the child's illness was dismissed. The mother was described by the staff as a bright and manipulative person, with good medical knowledge. She was a loner without any meaningful relationship with relatives or friends, including the father of the child. The communication with her own family was poor. She had bad memories about her childhood and reported that she had never received any help from her parents, who were affected by depression. When the child was 3 years old, her husband abandoned the family. After that she had a new partner and she had a second child. By that time, she presented an anxiety disorder and she was treated with pharmacological therapy. She was very concerned about the mother image that others had of her. Indeed, her greatest effort was to appear as a loving and skillful caregiver.

### Diagnosis of FDIA and outcome

Numerous inconsistencies were observed between the mother's and the teachers' description of the child and of his medical problems. No organic causes of M.'s symptoms were found; retrospectively the child's medical history appeared incoherent, with discrepancies between the described symptoms, the clinical observations and the results of multiple investigations. The mother was diagnosed as having a Mood and Anxiety Disorder, feelings of abandonment (parents' neglect, marital separation, poor social network). She never expressed doubts or concerns about her son's condition. She was very knowledgeable about all medical disorders reported for her child and attempted to manipulate the doctors and the hospital staff, requesting multiple invasive investigations.

For all these issues, the CNP suspected the mother's involvement in the child's illness and identified in the pattern of reported symptoms and behaviors a possible condition of FDIA, according to the above mentioned diagnostic criteria from the new classification of the DSM-5 [12].

Thus, he referred the case to the Juvenile Court, suggesting a psychiatric evaluation of the mother and the mother-child interaction. The child remained with his mother, under the surveillance and help of the social services, because she was the child's only caregiver after the father's abandonment. An individual psychotherapy and educational support for both the mother and the child were also instated.

## Second case report

### The victim

F. is a 12 years-old girl who was referred to our department of Adolescent Psychiatry after a 4 year history during which she experienced 18 separate episodes of hospitalization in 6 different Italian hospitals. When she arrived at our care she presented a wide repertory of medical symptoms, characterized by headache, abdominal pain, lipothymic episodes, seizures and a gait impairment. She is an only child. The mother is a housewife, the father is a truck drive, often working abroad for very long periods. The mother reported a difficult pregnancy with dystocia and a Caesarean section. F.'s referrals and hospitalizations started before she was 2 years old with a pediatric and ophthalmologic follow up requested for a case of strabismus with visual impairment. The frequency of referrals increased around the age of 8 years. At that time, she was referred to a hospital for a severe, drug-resistant throbbing headache, with bilateral localization in the parietal-occipital area. CT and fundus examination were normal. At the age of 13 she presented also frequent abdominal pain, in addition to the reported daily migraine for which she chronically assumed codeine plus paracetamol. She was referred several times to the same hospital for this reason. After one of these episodes, she underwent surgery for a case of suspected appendicitis. She also presented tonic-clonic seizure episodes. The described symptoms were always subjective or non-specific. All the symptoms were reported by the mother and were not verifiable at the time of the referral.

Over time the symptoms became more complex and articulated and were not described only by the mother but also by the young girl herself. At the age of 14, new symptoms were reported. The patient started to have difficulties in walking without help. She was referred for asthenia of the lower limbs, areas of hypoalgesia and also allodynia and migraine. She completely stopped walking, needing a wheelchair. All her labs and other diagnostic exams (metabolic panel -folate and B12-, MRI, angio-MRI, CT, autoimmune tests, EEG, 24-h holter monitoring and EKG) were normal, and no organic dysfunction or medical condition could be identified. The girl appeared perfectly healthy in spite of the reported symptoms. She was referred to our psychiatric day-hospital with the described clinical features.

### The perpetrator

F.'s mother is a 34 years old woman, described as very isolated person with poor social interactions. She is a housewife who used to spend all her time with her daughter and her mother in law, without any other social contact or interest. She was described by the husband as a passive person, completely lacking self-confidence. Her history was characterized by the premature death of her father when she was 15 years old. After that, she lived in indigence with her mother and a younger brother, with whom she did not maintain a good relation. She met her husband for the first time when she was 22 years old, and they got married in a haste because she had become pregnant with their only child. The husband reported that the child was the only link that kept them together. Indeed, he was more deeply attached to the child than to his wife. The attachment of the mother to the child was always characterized by a caregiving attitude with no great empathy or manifestation of feelings. During the periods of F.'s hospitalizations, she was extremely involved in the management of the child, which afforded to her the opportunity of networking with other people, including establishing significant relationships with the hospital staff. Furthermore, she did not appear to be worried for the increasing symptoms and illness of her daughter.

### Diagnosis of FDIA and outcome

During the hospitalization in our Department of Adolescent Psychiatry the girl was removed from her family for a period of 6 months. This window time allowed to better analyze the family dynamic and how the girl's behaviors changed while she was among her peers in several circumstances. A differential diagnosis was carried out between organic and somatoform symptoms. Thus, both a clinical investigation and a psychiatric evaluation were performed. The reported symptoms were investigated with a Clinical-EEG-polygraphic study that confirmed the non-epileptic nature of the seizures presented by the patient. A brain and spinal cord MRI and CT excluded lesions correlated with the motor impairment. Moreover, an Electromyogram (EMG) and Nerve Conduction Studies (normal) were performed in order to investigate a muscular or peripheral nervous system involvement in the strength and motor deficits. The psychiatric evaluation was based on direct interviews with the girl and the parents (both in couple and alone). Standardized tests were administered to the girl in order to evaluate the presence of personality disorders (MMPI), Anxiety and Mood disorders (STAY1-2; CDI; BDI), Dissociative and Somatoform Symptoms (SDQ; A-DES). The parent investigation evaluated the rate of parenting stress (PSI-Parent Stress Index) that can result in the Munchausen by proxy [13].

A denial of the disease emerged by the different self-reports, while the results of the SDQ−20 and A-DES were positive for dissociation and for somatoform dissociation. Additionally, the parent stress index of the mother resulted elevated. The therapeutic approach during the hospitalization consisted of individual psychotherapeutic sessions with the patient, and of parent training interventions. The patient attended also didactic activities, in line with her scholar level, and psychomotor rehabilitation therapy.

## Discussion

These case reports presented most of the main characteristics of fabricated or induced illness as underlined by the “Red Flags” for victims and perpetrators in the classical presentation of the FDIA (Tables 1, 2).

**Table 1:** FDIA red flags: the perpetrator.

Observations and investigation are inconsistent with the caregiver's report on the condition of the child
Vague and inconsistent details about child's medical history
Invasive diagnostic and surgery procedures are accepted without concern
The perpetrator shows medical knowledge
Requests are made for further interventions, procedures and second opinions
Attention and approval of medical staff are sought
Several medical appointments are missed
Previous history of psychiatric disorder
No relationships, family and marital problems

**Table 2:** FDIA red flags: the victim.

Atypical presentation of disorder
Tests and observations are normal
Medical problems don't respond to treatment
Symptoms and signs occur only in the caregiver's presence and disappear in his/her absence
Multiple hospitalizations and surgeries
Presence of multiple medical illness (e.g., mental disorder, microcephaly)
Occurrence of complications or of new pathology when the findings prove negative
Father is absent or not present in the life of the child

A feature common to both reports is the initial illness described in the children's history during their first period of life. Probably this experience elicited in the caregivers the concept and the feeling of an ill child (i.e., difficult pregnancy and delivery; strabismus; trauma accident).

A chronic state of fabricated illness and repeated hospitalizations was reported in both cases. The symptoms were prolonged and variable with time, without any positive or decisive finding in any of the diagnostic investigations. In both cases the clinical symptoms were purposely presented and constructed by the parent (mother), in order to maintain their caregiving role for an ill child [14]. The reported symptoms were mainly subjective or difficult to elicit in front of the medical staff, and indeed they were mainly described by the mothers. Epilepsy and headache are the most frequently fabricated illnesses to be reported [15, 16], even if the victims usually present multiple complaints [4].

Moreover, in both our cases the perpetrators were the mothers, who presented similarities both in their histories and in their child caregiving roles. Both reported a family history of neglect by their own parents and both showed psychiatric problems with anxiety and mood disorders, as described in the literature on the psychological profile of FDIA parents. In the family history, neuropsychiatric disorders were described in 35% of the cases reported in literature, in particular a familiarity for alcoholism was reported as well as mood and anxiety disorders [17].

Poor relationships with relatives and a poor social network appear to be environmental risk factors for FDIA conditions. The mothers, in both cases, were very isolated individuals with symbiotic links with their children. Also, conflictual relationships were reported in the parents' couples that were separated or experienced a conflictual marriage.

With regard to the medical condition of the children, the mothers did not show any concern in spite of being very well informed about the medical implications of those conditions. Furthermore, their behavior toward the hospital staff was very manipulative.

The two case reports differ for age and gender and present different characteristics in the relations with the perpetrator and the presentation of the chronic illness.

In the first case, it appears that the child learned to tolerate passively medical procedures in order to maintain a relationship with his mother, who in this case was the only available parent. Notwithstanding, he occasionally showed inappropriate and infantile behavior possibly related to the need to fabricate symptoms, which however he was unable to interrupt.

In the second case, the girl learned that the only way to create a relationship with the mother was through her symptoms and illness. Thus, when she grew up she shared and perpetuated the mother's modality of fabricating symptoms. Indeed, even when removed from her family, during the period of the hospitalization in our clinic, she maintained unmodified the presentation of her symptoms for a longtime. This mechanism has been described in the adult FDIA, as a Stockholm syndrome [18] or a follie-a-deux [19].

Moreover, she presented a conflictual family history where the link between her parents was mediated by her illness and by the care that she received. Thus, her symptoms were functional for the union of her family.

## Conclusion

FDIA is a serious form of child abuse with persistent and severe maltreatments, which can cause the child's death. An early diagnosis represents a challenge for the clinician and is essential in order to limit complications and to improve the outcome. The majority of FDIA reports in the literature are mainly focused on the history of child's abuse and few describe the “red flags” for an early identification, intervention, and possibly even prevention, of FDIA abuse [20]. It is debated whether a video surveillance of the perpetrator (resulting in the recording of abuse and maltreatment) is ethical and legal [21, 22]. Indeed, several cases of FDIA would not have been identified without the use of video surveillance [20]. Moreover, the DSM 5 [12] suggests that an accurate differential diagnosis should always be carried out between FDIA and case of Malingering.

## Ethics statement

This manuscript titled “Induced Illness in Children” had been revised and approved by the Ethics Committees of Sapienza University of Rome, Azienda Policlinico Umberto I (Protocol number:452/17). Informed consent was obtained from the guardians of the patients for the publication of these case reports.

## Author contributions

NF, VB, and GN conceptualized and designed the study, drafted the initial manuscript, and approved the final manuscript as submitted. MC and IA helped to collect data, reviewed the manuscript, and approved the final manuscript as submitted. RC and VG coordinated and supervised the work, critically reviewed the manuscript, and approved the final manuscript as submitted. All authors approved the final manuscript as submitted and agree to be accountable for all aspects of the work.

### Conflict of interest statement

The authors declare that the research was conducted in the absence of any commercial or financial relationships that could be construed as a potential conflict of interest.

## Abbreviations

MSbP: Münchausen syndrome by proxy
MRI: Magnetic Resonance Imaging
EEG: Electroencephalography
IQ: Intelligence Quotient
NMR: Nuclear Magnetic Resonance
CNP: Child Neuropsychiatrist
CT: Computed Tomography
EKG: electrocardiogram.

## References

[1] Pritchard C The Child Abusers: Research and Controversy. Maidenhead: Open University Press (2004).

[2] Craft AW Hall DMB. Munchausen syndrome by proxy and sudden infant death. Br Med J. (2004) 328:1309–12. 10.1136/bmj.328.7451.1309 15166071 PMC420178

[3] Fish E Bromfield L Higgins D. A New Name for Munchausen Syndrome by Proxy: Defining Fabricated or Induced Illness by Carers. NCPC (2005). Available online at: https://aifs.gov.au/cfca/publications/new-name-munchausen-syndrome-proxy-defining-fabric

[4] Sheridan MS. The deceit continues: an updated literature review of Munchausen Syndrome by proxy. Child Abuse Negl. (2003) 27:431–51. 10.1016/S0145-2134(03)00030-9 12686328

[5] Christie-Smith D Gartner C Understanding Munchausen Syndrome by Proxy. Special Report: Highlights of the 2004 Institute on Psychiatric Services (2004). Available online at https://psychiatryonline.org/10.1176/appi.ps.56.1.16 15637185

[6] Carter KE Izsak E Marlow J. Munchausen syndrome by proxy caused by ipecac poisoning. Pediatr. Emerg. Care (2006) 22:655–6. 10.1097/01.pec.0000227871.69309.d7 16983253

[7] Feldman KW Hickman RO. The central venous catheter as a source of medical chaos in Munchausen syndrome by proxy. J Pediatr Surg. (1998) 33:623–627. 9574764 10.1016/s0022-3468(98)90329-3

[8] Kumar R Bhuria J Mehta P Jain S Munchausen syndrome by proxy masquerading as pyoderma gangrenosum. Ind J Paediatr Dermatol. (2014) 15:123–4. 10.4103/2319-7250.143667

[9] Criddle L. Monsters in the closet: munchausen syndrome by proxy. CriticalCareNurse (2010) 30:46–55. 10.4037/ccn2010737 21123232

[10] Rosenberg D. Web of deceit: a literature review of Munchausen syndrome by proxy. Child Abuse Negl (1987) 11:547–63. 10.1016/0145-2134(87)90081-0 3322516

[11] Flaherty EG Macmillan HL Caregiver-fabricated illness in a child: a manifestation of child maltreatment; Committee ON Child Abuse And Neglect. Pediatrics (2013) 132:590–7. 10.1542/peds.2013-2045 23979088

[12] American Psychiatric Association Diagnostic and Statistical Manual of Mental Disorders, 5th Edn Arlington, VA: American Psychiatric Publishing (2013).

[13] Wrennall L. Munchausen Syndrome by Proxy/Fabricated and Induced Illness: does the diagnosis serve economic vested interests, rather than the interests of children? Med Hypotheses (2007) 68:960–6. 10.1016/j.mehy.2006.10.016 17141423

[14] Ferrara P Vitelli O Bottaro G Gatto A Liberatore P Binetti P. Factitious disorders and Munchausen syndrome: the tip of the iceberg. J Child Health Care (2013) 17:366–74. 10.1177/1367493512462262 23411659

[15] Doughty K Rood C Patel A Thackeray JD Brink FW. Neurological manifestations of medical child abuse. Pediatr. Neurol (2016) 54:22–8. 10.1016/j.pediatrneurol.2015.09.010 26608710

[16] Meadow R. Munchausen syndrome by proxy: the hinterland of child abuse. Lancet (1977) 2:343–5. 10.1016/S0140-6736(77)91497-0 69945

[17] Libow JA Child and adolescent illness falsification. Pediatrics (2000) 105:336–42. 10.1542/peds.105.2.336 10654952

[18] Spuijbroek EJ Blom N Braam AW Kahn DA. Stockholm syndrome manifestation of Munchausen: an eye-catching misnomer. J Psychiatr Pract. (2012) 18:296–303. 10.1097/01.pra.0000416021.68462.f3 22805905

[19] Günter M. Induction, identification or folie à deux? Psychodynamics and genesis of Munchausen syndromes by proxy and false allegations of sexual abuse in adolescents. Med Law (1998) 17:359–79. 9922627

[20] Wahi MM Stone KV Chance M Miller CE Patient-centered medicine and prevention of munchausen syndrome by proxy. In: Sayligil O editor. Patient Centered Medicine. InTech (2017). Available online at: https://www.intechopen.com/books/patient-centered-medicine/patient-centered-medicine-and-prevention-of-munchausen-syndrome-by-proxy

[21] Bauer KA Covert video surveillance of parents suspected of child abuse: the British experience and alternative approaches. Theor Med Bioeth (2005) 25:311–27. 10.1007/s11017-004-3145-7 15637948

[22] Byard RW Burnell RH Covert video surveillance in Munchausen syndrome by proxy. Ethical compromise or essential technique? Med J Aust. (1994) 160: 352–6. 8133820

